# High resolution Tibetan Plateau regional reanalysis 1961-present

**DOI:** 10.1038/s41597-024-03282-4

**Published:** 2024-05-03

**Authors:** Peifeng Zhou, Jianping Tang, Mengnan Ma, Dabin Ji, Jiancheng Shi

**Affiliations:** 1 Key Laboratory of Mesoscale Severe Weather/Ministry of Education, Nanjing University, Nanjing, 210023 China; 2 School of Atmospheric Sciences, Nanjing University, Nanjing, 210023 China; 3 State Key Laboratory of Remote Sensing Science, Aerospace Information Research Institute, Chinese Academy of Sciences, Beijing, 100101 China; 4 National Space Science Center, Chinese Academy of Sciences, Beijing, 100190 China

**Keywords:** Atmospheric dynamics, Climate and Earth system modelling

## Abstract

With the rapid global warming in recent decades, the Tibetan Plateau (TP) has suffered severe impacts, such as glacier retreat, glacial lake expansion, and permafrost degradation, which threaten the lives and properties of the local and downstream populations. Regional Reanalysis (RR) is vital for TP due to the limitations of observations. In this work, a 62-year (1961–2022) long atmospheric regional reanalysis with spatial resolution of 9 km (convective gray-zone scale) and temporal resolution of 1 hour over the TP (TPRR) was developed using the Weather Research and Forecasting (WRF) model, combined with re-initialization method, spectral nudging (SN), and several optimizations. TPRR is forced by ERA5 at hourly intervals. TPRR outperforms ERA5, realistically capturing climatological characteristics and seasonal variations of precipitation and T2m (air temperature at 2m above ground level). Moreover, TPRR better reproduces the frequency and intensity of precipitation, as well as the diurnal cycle of precipitation. This study also quantifies the wetting trend of 0.0071 mm/year over the TP amid global warming using TPRR.

## Background & Summary

The Tibetan Plateau (TP) has the largest number of glaciers outside the polar regions, and is the source of principal rivers in Asia, such as the Yangtze River, the Yellow River, and the Ganges River, known as the “Water tower of Asian”^1–3^. In addition, TP is the highest and most extensive highland in the world, with unique complex terrain and distinctive underlying surface, exerting a great influence on regional and global climate through its thermal and dynamical forcing mechanisms^4–7^. In recent decades, TP has undergone severe changes due to global warming. Observations indicate that the warming rate over the TP has exceeded twice the global average during the same period and the warming rate is continuously increasing^2,4,8–11^. Glacier retreat, glacial lake expansion, and permafrost degradation^12–16^, which result from the rapid warming increase the frequency and intensity of natural disasters^17–19^, threatening large local and downstream Asian populations. Therefore, an accurate long-term climate dataset with high spatiotemporal resolution is crucial for studying the response of TP to global warming and adapting for natural disasters.

Precipitation and surface air temperature are the most important and basic meteorological elements, which can be generally measured in three ways: *in-situ* observations, radar observations, and satellite remote sensing observations. The *in-situ* observations can provide the most direct estimates of precipitation and air temperature at 2 m above ground level (AGL) (T2m). Nevertheless, the distribution of the *in-situ* stations over the TP is spare and uneven. Owing to the complex terrain and harsh environmental conditions, most *in-situ* stations are often located in the valley of eastern and central TP^20–22^. Weather radars suffer from beam blockages and range-dependent biases in mountainous areas^23^. Satellite precipitation products provide estimates of precipitation indirectly, which is affected by observations, sampling, retrieval algorithms, and bias correction processes^24,25^. Moreover, satellite products show poor ability in capturing solid precipitation^26^ and uncertainties in mountain areas^27,28^.

Recently, global reanalysis (GR) has been applied to TP, such as ERA-Interim, ERA5, and JRA-55^29–34^. Reanalysis products are characterized by temporal stability and spatial continuity, which can solve the problem arising from the inhomogeneous distribution of observation stations. However, GR with coarse resolution has biases over the TP due to inaccurate representation of its complex terrain, such as wet and cold biases in ERA5 with a resolution of 0.25° × 0.25°. Regional reanalysis (RR) which focuses on a specific region exhibits superiority over GR in describing climate and its variability with higher resolution^35,36^. There are several methods applied in RR, including continuous nudging method, three-dimensional variational data assimilation (3DVAR), four-dimension variational data assimilation (4DVAR), and re-initialization method^35,37–44^. Based on the above methods, RR has been applied in many regions; for example, North American Regional Reanalysis (NARR)^37^ is applied in North America using 3DVAR, East Asia Reanalysis System (EARS)^44^ is applied in East Asia using 4DVAR/3DVAR on surface/upper observations, the Arctic System Reanalysis version 2 (ASRv2)^35^ is applied in Arctic using 3DVAR, COSMO-REA2^40^ is applied in Central Europe using continuous nudging method, and the Indian Monsoon Data Assimilation and Analysis (IMDAA)^41^ is applied in the Indian subcontinent using 4DVAR.

To strike the balance between huge computational cost and comparatively accurate performance, the gray-zone scale (about 9 km) is widely used in regional climate simulation and regional reanalysis over the TP^45–47^. The High Asia Reanalysis (HAR)^38^ and its second version HARv2^42^ have been developed over the TP with the resolution of 10 km in the inner domain, using Grell 3D^48,49^ and KF-CuP^50^ cumulus parameterization schemes (CPSs), respectively. Ou *et al*.^51^ demonstrate that a simulation without a CPS describes the precipitation diurnal cycle more accurately than that with a CPS at the gray-zone scale. Several studies showed that HARv2 overestimated precipitation in the Minjiang River basin^52^ (located at eastern margin of the TP) and Qilian Mountains^53^ (located at the northeastern margin of the TP), closed CPS at gray-zone scale may contribute to reducing the wet bias. Since corrected the initial snow depth in ERA5 using Japanese 55-year Reanalysis (JAR-55), the simulation of T2m has been greatly improved^54^. Yan *et al*.^55^ demonstrated GLDAS performed better than JRA-55 in the simulation of snow cover fraction over the TP. Sun *et al*.^56^ indicated that snowfall of GLDAS showed closer relationship to observation than that of HARv2 which overestimated snowfall especially in spring. Therefore, using the snow products of GLDAS to correct the initial forcing may improve the performance of simulation. Hence, based on several methods and optimizations, the goals of this study were (1) to develop a 62-year (1961–2022) hourly atmospheric regional reanalysis with spatial resolution of 9 km over the TP (TPRR) using the Weather Research and Forecasting (WRF) model; and (2) to apply TPRR to assess the wetting trend under global warming over the TP. The long-term TPRR with high spatial and temporal resolution can enhance the understanding of climate change over the TP and provide reliable data support for more refined simulation in the future.

## Methods

### Model configurations

**Fig. 1:**
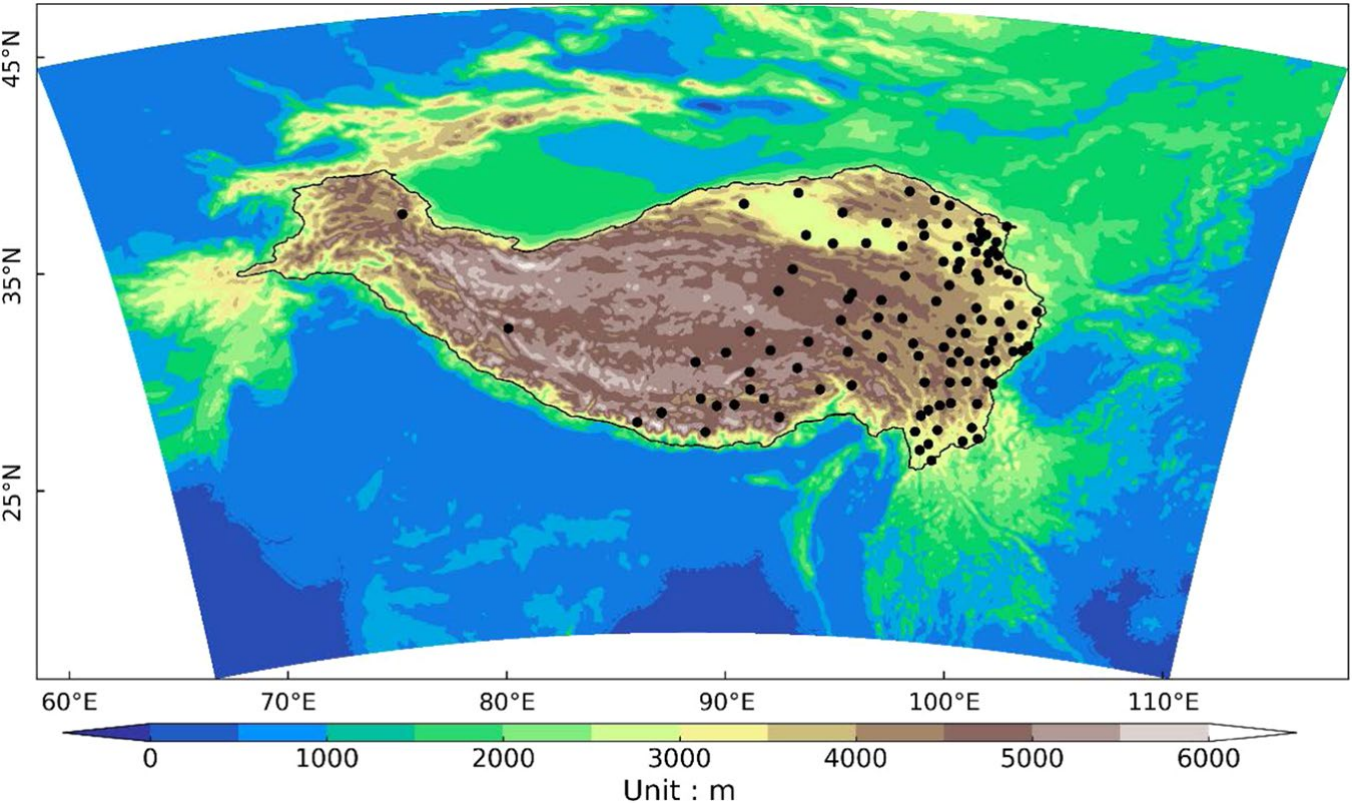
Domain and topography of the WRF experiment (unit: m). Black dots represent the distribution of *in-situ* stations, and the TP is framed with black lines.

The state-of-the-art non-hydrostatic WRF model (version 4.2)^57^ was used in this study. The model domain was configured at 9 km grid spacing and centered at 33.0° N, 87.5° E, with 530 grid points in the east-west direction and 360 grid points in the north-south direction, covering the TP and its surrounding areas (Fig. 1). There are 49 uneven levels distributed vertically with the model top at 10 hPa. Based on the previous studies^46,51,58–60^ over the TP, the following physical parameterization schemes were used in this study: RRTMG long-wave and short-wave radiative transfer scheme^61^, the Unified Noah land surface model (NOAH)^62^, Mellor-Yamada Nakanishi and Niino Level 2.5 (MYNN2.5) planetary boundary layer (PBL) parameterization^63^ and Thompson microphysics parameterization scheme^64^. According to several studies^51,65,66^, the cumulus parameterization is switched off.

### Input data

The initial and lateral boundary conditions are provided by the ECMWF fifth generation atmospheric reanalysis ERA5^32,67,68^, which has the temporal resolution of 1 hour and spatial resolution of 0.25°. Nevertheless, ERA5 clearly overestimates snow depth (SD), snow cover (SC), and snow water equivalent (SWE) over the TP, which may be attributed to the absence of the Interactive Multisensor Snow and Ice Mapping System (IMS) SC products in the assimilation above 1500 m^69,70^. In this study, the initial fields of SD and SWE in ERA5 are replaced by those from the Global Land Data Assimilation System (GLDAS)^71,72^, which is closer to *in-situ* observations and IMS^73^. In addition, the default setup uses the sea surface temperature (SST) in the closest oceanic grid cell as surface temperature for lake, therefore, surface temperature for lake in TPRR is replaced by daily-averaged surface air temperature by the avg_tsfc.exe module in WRF preprocess.

### Workflow to generate TPRR

**Fig. 2:**
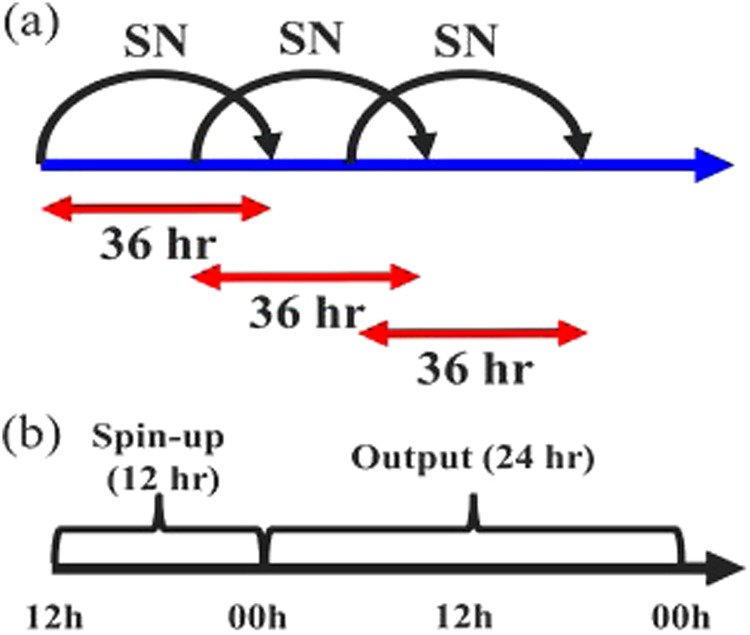
Workflow of TPRR product, including each short-term run (**b**) and their combination (**a**).

The method that subdivides the long-term continuous integration into short ones can alleviate the growth of systematic errors in long-term integrations, which is the so-called re-initialization. Maussion *et al*.^38^ and Wang *et al*.^42^ conducted HAR and HARv2 using re-initialization, and both datasets show good performance in describing the climate over the TP. In this study, the re-initialization strategy was adopted, and the spectral nudging (SN) method^74^ which is considered as an indirect assimilation method and can prevent the simulation from drifting away from the forcing, was applied to wind fields above planetary boundary layer (PBL). The workflow for producing TPRR is presented in Fig. 2. Each short run starts from 12:00 UTC and is integrated for 36 hours, with the first 12 hours treated as spin-up time and the remaining 24-hour output combined into the long-term TPRR. For the potential users of TPRR (atmospheric scientists, hydrological scientists, etc.), post processing is applied to the output of WRF for ease of use. TPRR provides common surface and upper-air isobaric meteorological variables, separated by per variable, per year and per time aggregation (hourly and daily).

## Data Records

The high-spatiotemporal-resolution (9 km,1 h) TPRR^75^ presented and analyzed in this article is freely available at 10.11888/Atmos.tpdc.300821. The database format is NETCDF version 4, and the total volume of the data files is 4.25 TB. Daily total precipitable water (TPW) integrated from bottom to top of model atmosphere and hourly precipitation, T2m, u-component and v-component wind at 10 m AGL (U10, V10), specific humidity at 2 m AGL (Q2), and the terms involved in surface energy balance on the ground are provided in TPRR. TPRR is currently available by contacting the author for permission, and will be accessed freely at this location soon.

## Technical Validation

Several validation datasets including *in-situ* observations and satellite precipitation product are used to evaluate the performance of TPRR. The daily *in-situ* observations are provided by the data service center at China Meteorology Administration^76^ (CMA), including daily T2m and daily precipitation. The station observations have gone through quality control and standard normal homogeneity test^77^ (SNHT). There are 111 gauge stations over the TP which can provide long-term observations from 1961 to 2022 and most of them are located in eastern TP (Fig. 1). The Integrated Multi-satellite Retrievals for GPM version 6^78^ (IMERG) with high spatial (0.1° × 0.1°) and temporal (30 minutes) resolutions is used in this study to evaluate the diurnal cycle of simulated precipitation. IMERG is considered as one of the most superior precipitation products that can provide reliable precipitation characteristics over the TP^79–82^. Precipitation data in IMERG from 2001 to 2020 is used in this study. To evaluate the performance of TPRR, TPRR is interpolated to *in-situ* observations and IMERG using the nearest interpolation method, respectively. Due to the difference in terrain height between TPRR and station locations, the annual mean lapse rate (LR)^83^ over the TP is used to correct biases of T2m in TPRR after interpolation. Among the meteorological variables in TPRR, precipitation and T2m is evaluated in this section.

In this study, two statistical metrics are selected to validate the estimates of TPRR, which are root mean square error (RMSE) and correlation coefficient (CORR). The equations for these parameters are expressed as follows:

$${\rm{RMSE}}=\sqrt{\frac{1}{M}\mathop{\sum }\limits_{1}^{M}{({y}_{i}^{est}-{y}_{i}^{obs})}^{2}}$$

where

$${\rm{C}}{\rm{O}}{\rm{R}}{\rm{R}}=\frac{{\sum }_{1}^{M}[({y}_{i}^{obs}-\bar{{y}^{obs}})({y}_{i}^{est}-\bar{{y}^{est}})]}{\sqrt{{\sum }_{1}^{M}{({y}_{i}^{obs}-\bar{{y}^{obs}})}^{2}{\sum }_{1}^{M}{({{y}_{i}^{est}}_{i}-\bar{{y}^{est}})}^{2}}}$$

and ${y}_{i}^{obs}$ are the estimations and observations, respectively, $\overline{{y}^{est}}$ and $\overline{{y}^{obs}}$ are the means of estimations and observations, respectively, the M is the number of stations or the number of times.

### Evaluation of precipitation and T2m

**Fig. 3:**
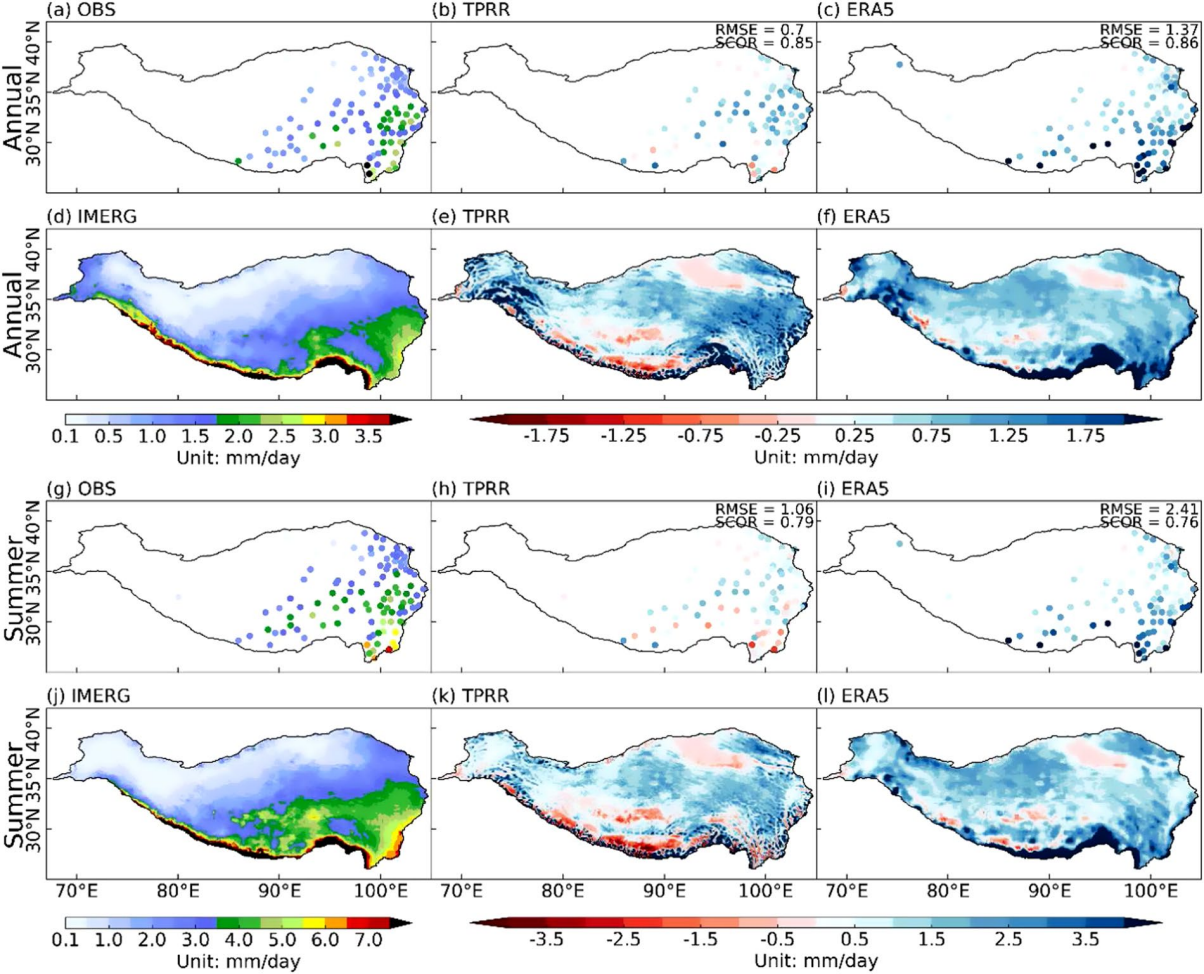
The 62-year (1961–2022) and 20-year (2001–2020) averaged annual (**a,d**) and summer (**g,j**) mean daily precipitation from *in-situ* observations and IMERG, respectively, and the differences between TPRR (**b,h**), ERA5 (**c,i**) and *in-situ* observations (interpolated to *in-situ* observations), and the differences between TPRR (**e,k**), ERA5 (**f,l**) and IMERG (interpolated to IMERG), unit: mm/day.

Figure 3 shows the 62-year (1961–2022) and 20-year (2001–2020) averaged annual and summer mean daily precipitation for TPRR and ERA5 against *in-situ* observations and IMERG, respectively. Compared to the *in-situ* observations, TPRR can well simulate the distribution of annual and summer mean precipitation with the spatial CORR (SCOR) of 0.85 and 0.79 and RMSE of 0.7 mm/day and 1.06 mm/day, respectively. Compared to the vast wet biases in ERA5 throughout the year and summer especially over the eastern and central TP, TPRR can largely reduce the wet biases. It can be found that annual and summer mean precipitation gradually decrease from southeast to northwest, with the maxima occurring at the southern edge of TP, as shown in Fig. 3d,j. Most precipitation occurs in summer, which is more than twice that of the annual precipitation. Compared to IMERG, wet biases in ERA5 exist in most regions of TP (Fig. 3f,l) at both annual and seasonal scales, especially at the southern edge. By contrast, TPRR reduces the wet biases over the whole TP, though dry biases exist over the central TP (Fig. 3e,k). In addition, TPRR shows more spatial details which are related to topography characteristics (Fig. 1), especially at the southern edge of TP and over the southeastern TP.

**Fig. 4:**
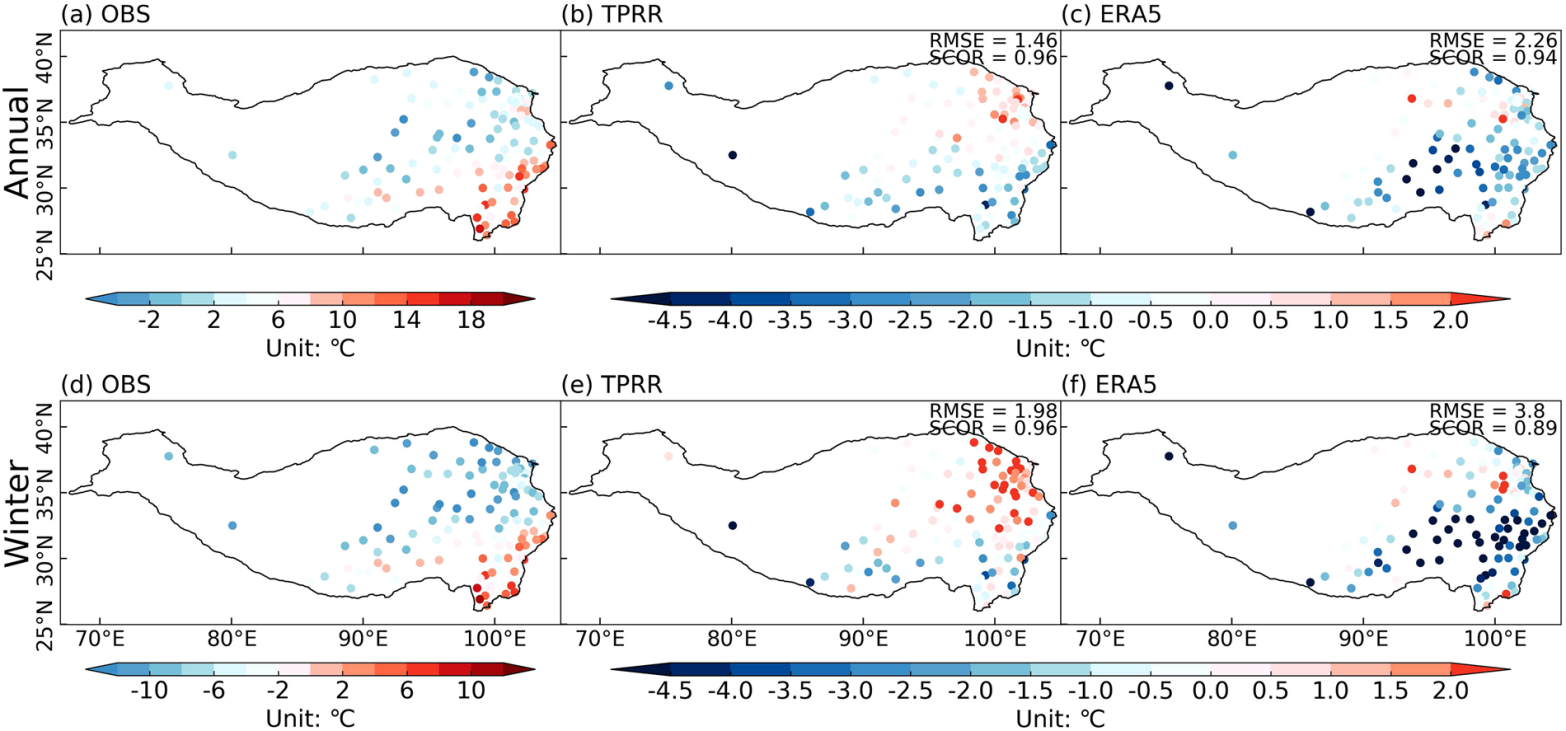
The 62-year (1961–2022) averaged annual (**a**) and winter (**d**) mean T2m from *in-situ* observations, and the differences between TPRR (**b,e**), ERA5 (**c,f**) and *in-situ* observations (interpolated to *in-situ* observations), unit: °C.

Figure 4 shows the 62-year (1961–2022) averaged annual and winter mean T2m from *in-situ* observations and the differences between TPRR, ERA5, and observations. Based on *in-situ* observations, annual and winter mean T2m decrease from southeast to northwest gradually (Fig. 4a,d). ERA5 (Fig. 4c,f) shows large cold biases over the eastern and central TP which can reach more than 5 °C, especially in winter. TPRR reduces these cold biases enormously over the whole TP, with RMSE of 1.46 °C (1.98 °C) and SCOR of 0.96 (0.96) for annual (winter) mean T2m.

**Fig. 5:**
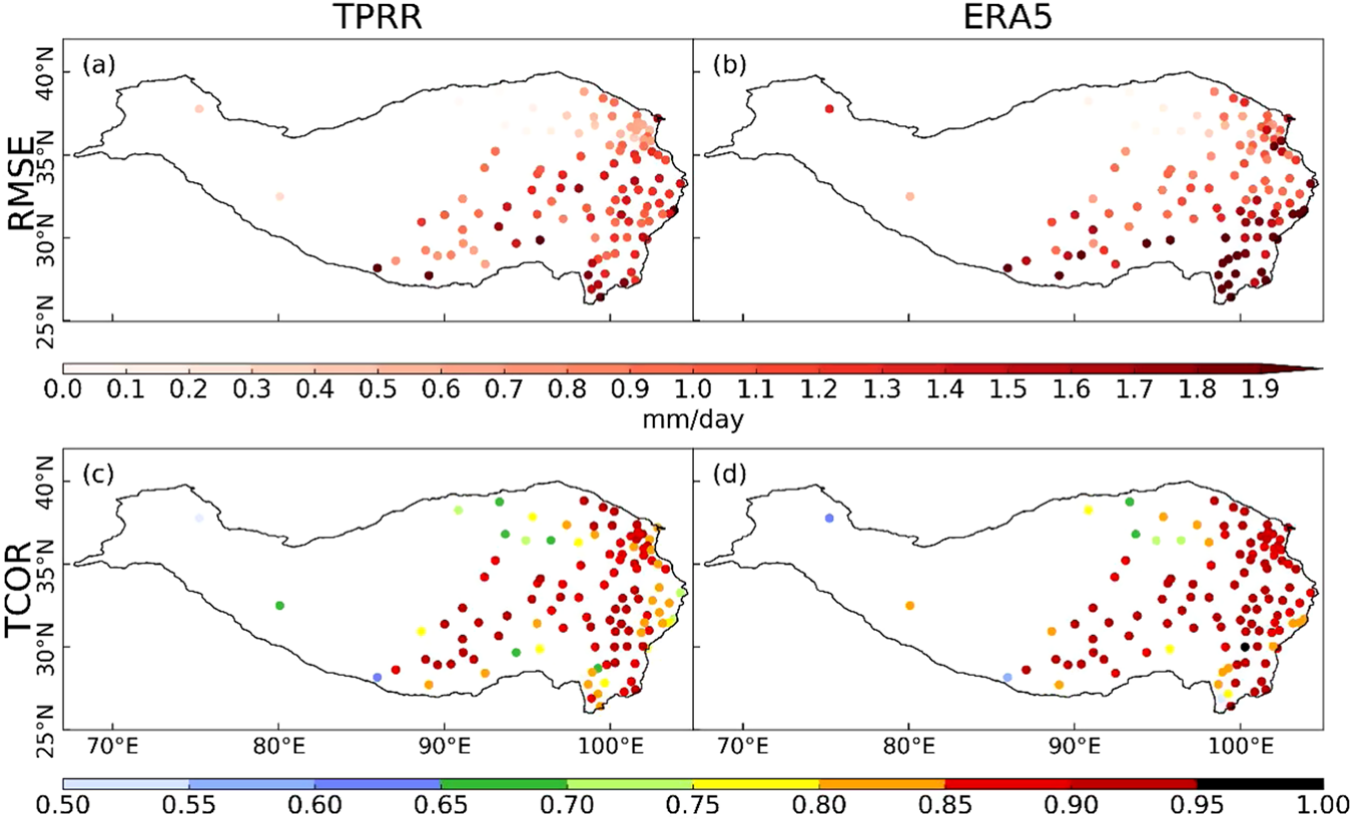
The spatial distribution of RMSEs (row 1) and TCORs (row 2) of monthly averaged precipitation from TPRR and ERA5 compared with *in-situ* observations, unit of RMSEs: mm/day.

**Fig. 6:**
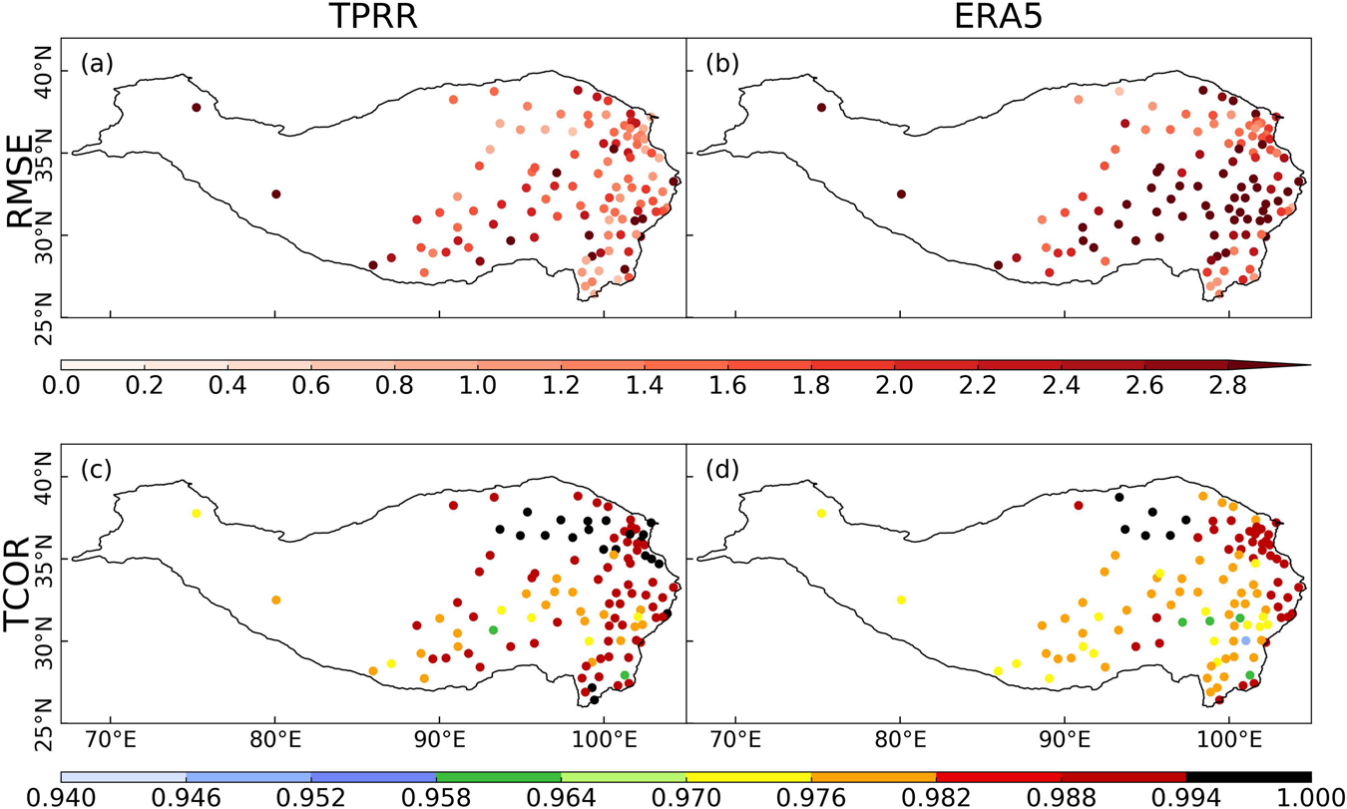
same as Fig. 5 but for T2m, unit of RMSEs: °C.

Figures 5, 6 depict RMSEs and temporal CORRs (TCORs) of monthly mean precipitation and T2m at each station between TPRR, ERA5, and *in-situ* observations for the period 1961–2022. For precipitation, TPRR can largely reduce the RMSEs that existed in ERA5, especially over the southeastern TP. The TCORs of TPRR are close to those of ERA5, except for several stations over the eastern edge of TP where the TCORs of these stations in TPRR are about 0.05 lower than those in ERA5. For T2m, the RMSEs of most stations in TPRR are lower than 2.8 °C, while those over the central TP in ERA5 are constantly larger than 2.8 °C. In addition, the TCORs of most stations over the TP in TPRR are slightly raised compared to ERA5.

**Fig. 7:**
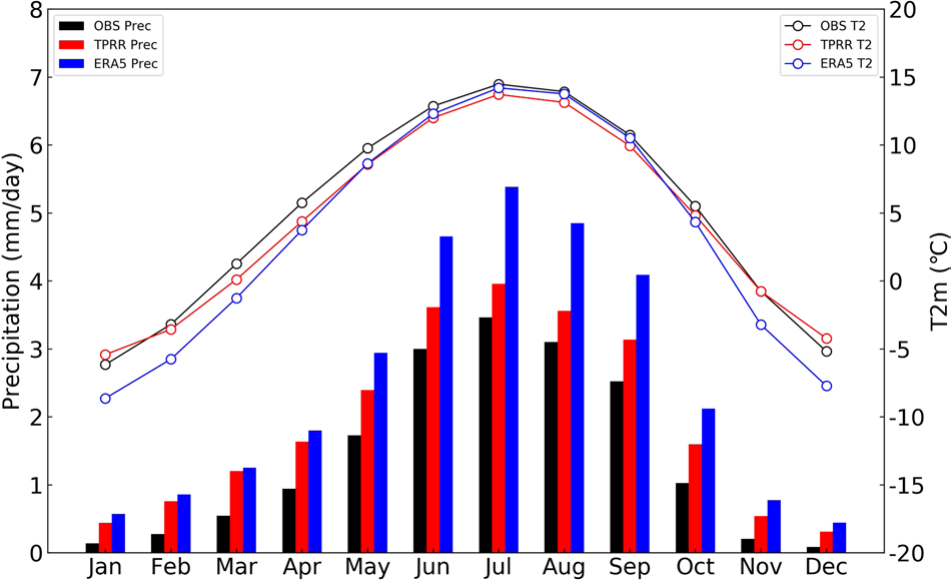
Seasonal cycle of 62-year (1961–2022) monthly averaged precipitation (histogram, unit: mm/day) and T2m (line chart, unit: °C) averaged over TP.

The monthly variations of precipitation and T2m averaged over the TP are shown in Fig. 7. Under the joint effect of Westerlies and Asian Monsoon, the precipitation over the TP mostly occurs in summer. ERA5 significantly overestimates monthly precipitation, while TPRR greatly reduces the wet biases, especially in summer. For T2m, ERA5 tends to produce large cold biases in cold seasons and TPRR can largely reduce these cold biases. TPRR shows slightly poorer performance than ERA5 in summer for T2m.

In general, TPRR shows superior performance than ERA5 in capturing the spatial distribution, monthly variability, and seasonal variation of precipitation and T2m, with better represented spatial heterogeneity.

### Daily precipitation frequency and intensity

**Fig. 8:**
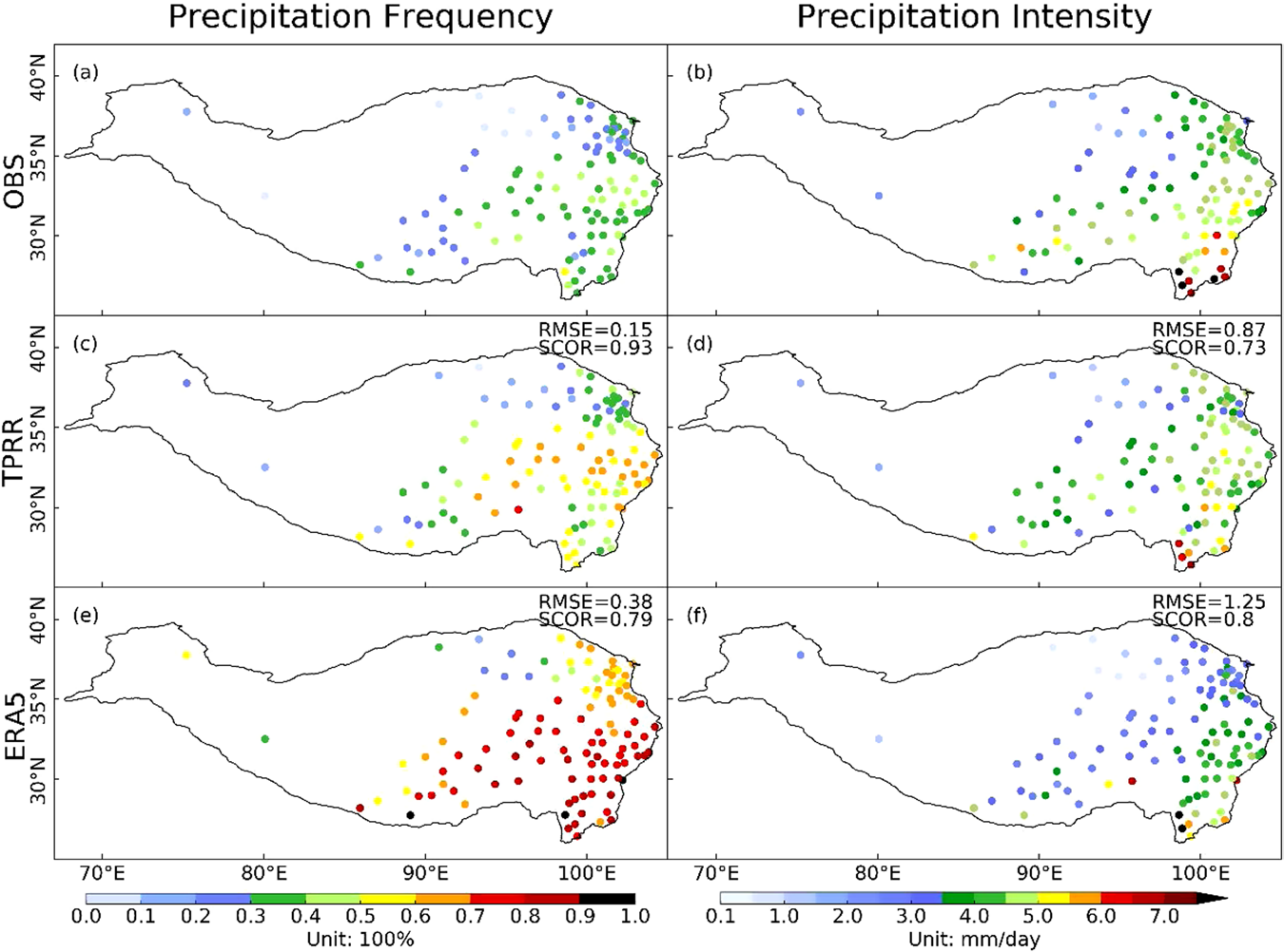
Spatial distribution of 62-year averaged daily precipitation frequencies (column 1, unit: 100%) and intensities (column 2, unit: mm/day) from *in-situ* observations (row 1), TPRR (row 2) and ERA5 (row 3).

A day with an accumulated precipitation larger than 0.1 mm is defined as a rainy day. The daily precipitation frequency and intensity are represented as the percentage of rainy days over all days and the precipitation amount averaged over these rainy days, respectively. The 62-year mean daily precipitation frequencies and intensities from *in-situ* observations, TPRR, and ERA5 are calculated and shown in Fig. 8. From the observation, the precipitation frequency gradually decreases from southeast to northwest with the highest frequency over the southeast TP which is below 50%. Although ERA5 reproduces the distribution of precipitation frequency with a SCOR of 0.79, it simulates more daily precipitation events especially over the southeastern TP with a frequency fraction above 80%. TPRR simulates precipitation frequencies more closely with those in *in-situ* observations over the southeastern TP where the overestimation is the most severe in ERA5. The strong precipitation intensities (above 5 mm/day) are observed over the southeastern TP and decrease to the northwest gradually. TPRR well reproduces the spatial distribution of precipitation intensity, with RMSE of 0.87 mm/day and SCOR of 0.73. The overestimation of precipitation amount and daily precipitation frequency over the southeastern TP in ERA5 is accompanied by a slight underestimation of precipitation intensity. In other areas of TP, ERA5 underestimates the precipitation intensity by more than 1 mm/day compared to *in-situ* observations.

**Fig. 9:**
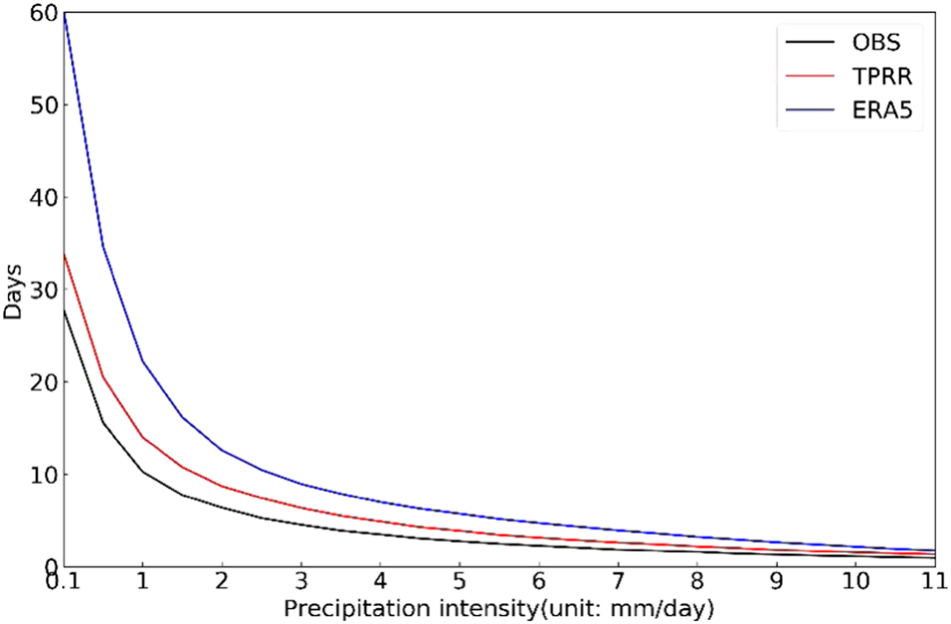
The number of annual averaged precipitation days binned by intensity (unit: mm/day) from *in-situ* observations, TPRR and ERA5.

Figure 9 shows the annual precipitation days at different precipitation intensities (0.5 mm/day interval) from *in-situ* observations, TPRR, and ERA5 over the TP. It can be found that TPRR describes the precipitation days for all intensities more accurately than ERA5, especially for intensities below 2 mm/day.

### Diurnal cycle of precipitation

**Fig. 10:**
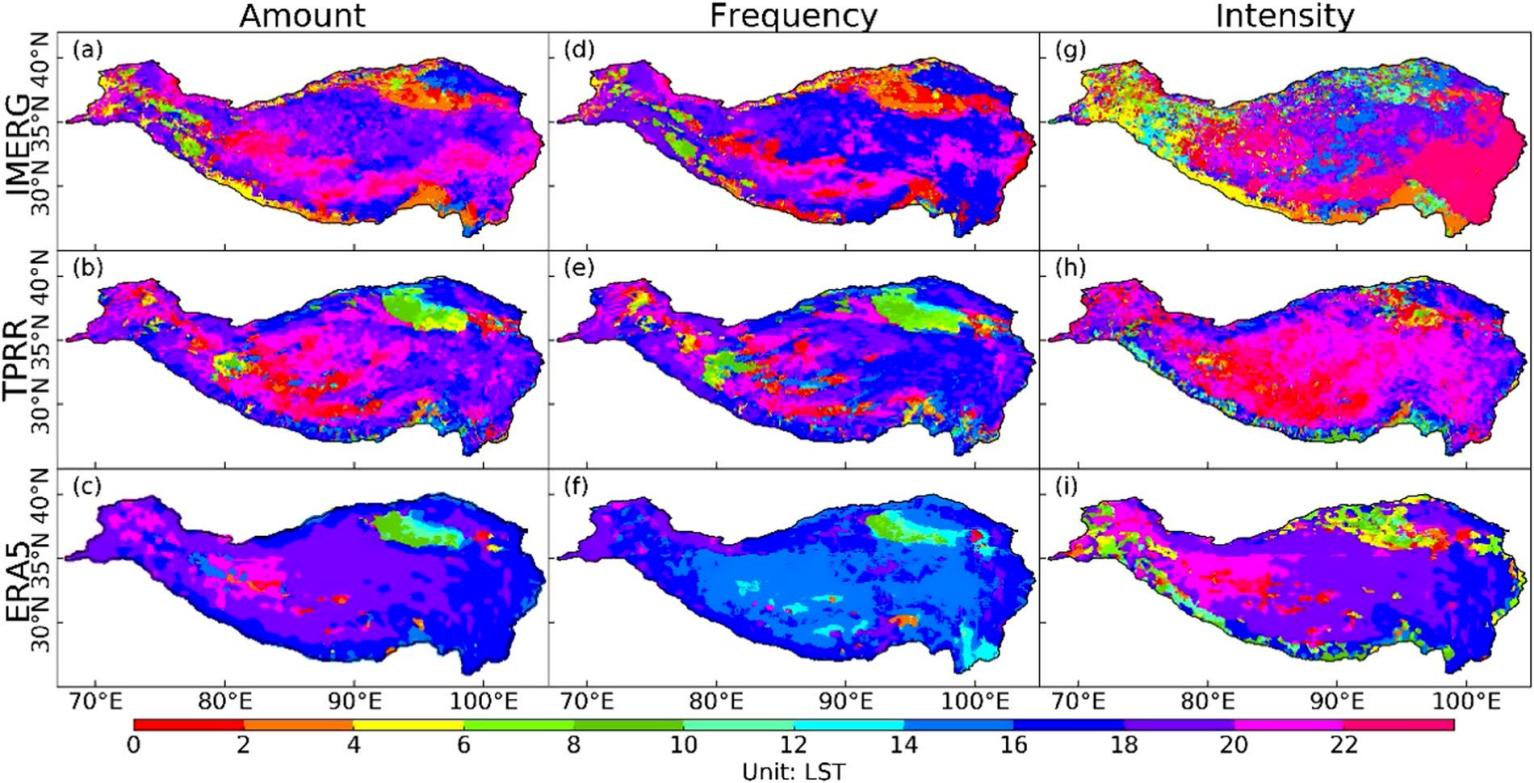
20-year (2001–2020) diurnal peak time (LST, unit: hour) of precipitation amount (column 1), frequency (column 2), and intensity (column 3) in IMERG (row 1), TPRR (row 2) and ERA5 (row 3).

The IMERG satellite precipitation product is used as the reference when evaluating the performance of diurnal precipitation in TPRR and ERA5. Figure 10 shows the peak time (LST, Local Standard Time, which is Universal Time minus 8 hours in this study) of diurnal precipitation amount, frequency, and intensity during 2001–2020 from IMERG, TPRR, and ERA5. With the improved representation of topography, TPRR has better depicted the peak time of diurnal precipitation amount, frequency, and intensity with more spatial details. Based on IMERG, the maximum precipitation amount at hourly scale occurs from dusk to late evening (1800–2400LST) in most regions of TP, while it tends to occur from nighttime to early morning (0200–0600LST) over Qaidam Basin and in regions with relatively low altitude. TPRR can generally reproduce the peak time of precipitation amount over the central and eastern TP. While it simulates the peak time about 2-hour later/earlier than IMERG over the central/eastern TP. Significantly advanced peak time of precipitation amount can be found in ERA5 over the central and eastern TP, where the maximum precipitation amount occurs in 1800–2000LST/1600–1800LST over the central/eastern TP. It can be found that TPRR better reproduces the peak time of precipitation amount over the whole TP. For precipitation frequency, based on IMERG, its spatial distribution is similar to that of precipitation amount, but is about 2 hours ahead of the precipitation amount peak. The peak time of precipitation frequency can be well reproduced in TPRR, especially over the central TP, but a 2-hour earlier peak for frequency than that in IMERG over the southeastern TP occurs in TPRR. Most of the overestimated precipitation frequency in ERA5 (Fig. 8e) occur mainly in the early afternoon (1400–1600LST), which may be attributed to the excessive convective precipitation. For precipitation intensity which is obtained by dividing amount by frequency, the peak time in IMERG occurs in late evening (2200-0000LST) over the eastern TP but from afternoon to evening (1400–2200LST) over the central TP. TPRR shows an earlier/later peak time over the eastern/central TP. The spatial distribution of intensity in ERA5 is similar to that of amount. ERA5 simulates an approximately 6-hour/2-hour earlier peak over the eastern/central TP.

### Wetting trend over TP

**Fig. 11:**
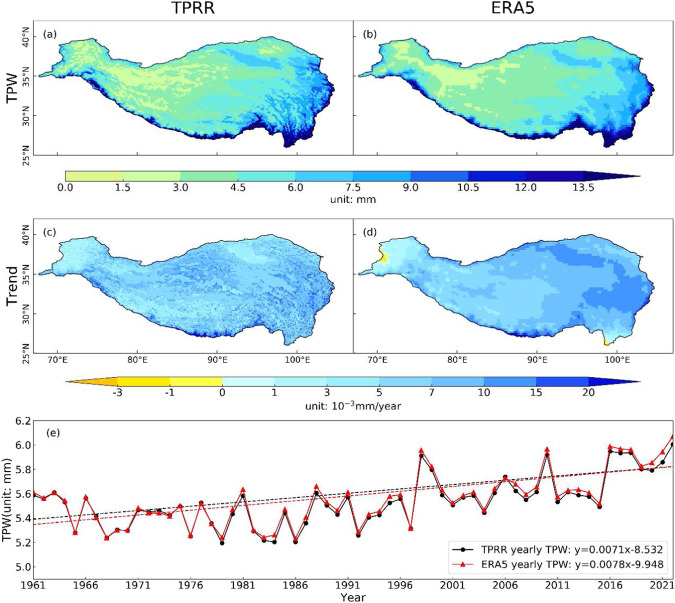
Spatial distribution of climatological mean state (row 1, unit: mm) and trend (row 2, unit: 10^−3^mm/year) of 62-year mean TPW from TPRR (column 1) and ERA5 (column 2) and times series of annual mean TPW (row 3, unit: mm) from TPRR (black dots) and ERA5 (red triangles).

Several studies^84–86^, with different datasets utilized, present a wetting trend over the TP. In this study, TPRR and ERA5 are used to quantify the wetting trend. Figure 11 shows the spatial distribution of the climatological mean state, trend and the time series of TPW from TPRR and ERA5. TPW is defined as the vertical integration of water vapor in the atmospheric columns from the surface to the model top, and is calculated as follows^87^:

1 $$TPW=-\frac{1}{g}{\int }_{{p}_{sfc}}^{{p}_{top}}qdp$$

where *g* is the gravity acceleration, *q* is the specific humidity, *p* is the pressure, *p*_*top*_ and *p*_*sfc*_ are defined as the top and bottom pressure of simulation.

Based on the spatial pattern of climatological mean TPW, TPW decreases gradually to the northwest with maximum values above 13.5 mm over the southeastern TP (Fig. 11a). The wetting trend can be found over the whole TP, especially at the southern edge of TP and over the eastern TP, with the maxima reaching about 0.02 mm/year (Fig. 11c). It is found in TPRR that the regions with relatively low altitude exhibits a faster wetting trend. When averaged over the whole TP, TPRR (0.0071 mm/year) shows a similar wetting trend to ERA5 (0.0078 mm/year) (Fig. 11e).

## Data Availability

The TPRR setup is available through the Weather Research and Forecasting (WRF) model. WRF is provided through a git repository (https://github.com/wrf-model/WRF/tags) available at the model’s website (https://www2.mmm.ucar.edu/wrf/users). The users can download the source code of WRF model in the git repository anonymously. The Python scripts used in this study for data post processing can be available through the following git repository: https://github.com/PayphoneChoou/TPRR_CODE.
